# Effects of standard and total two-field lymph node dissection on prognosis of patients undergoing Esophagectomy

**DOI:** 10.12669/pjms.38.4.4031

**Published:** 2022

**Authors:** Qiang Guo, Hefei Li, Haibo Wang, Duo Zhang, Yonghui Li

**Affiliations:** 1 Qiang Guo, Department of Thoracic Surgery, Affiliated Hospital of Hebei University, Baoding, Hebei 071000, P.R. China; 2 Hefei Li, Department of Thoracic Surgery, Affiliated Hospital of Hebei University, Baoding, Hebei 071000, P.R. China; 3 Haibo Wang, Department of Thoracic Surgery, Affiliated Hospital of Hebei University, Baoding, Hebei 071000, P.R. China; 4 Duo Zhang, Department of Thoracic Surgery, Affiliated Hospital of Hebei University, Baoding, Hebei 071000, P.R. China; 5 Yonghui Li, Department of Thoracic Surgery, Affiliated Hospital of Hebei University, Baoding, Hebei 071000, P.R. China

**Keywords:** Esophageal carcinoma, Esophagectomy, Lymph node dissection, Survival rate

## Abstract

**Objectives:**

To investigate the effects of standard two-field lymph node dissection (2FLND) and total 2FLND on the short-term and long-term clinical efficacy and complications of patients undergoing esophagectomy.

**Methods:**

The clinical data of 268 patients undergoing radical Ivor-Lewis esophagectomy in our hospital from January 2008 to November 2015 were analyzed retrospectively. According to different methods of lymph node dissection (LND), the patients were divided into standard 2FLND group (n = 121) and total 2FLND group (n = 147). The LND status, postoperative complications, survival rate and lymph node recurrence of the two groups were analyzed.

**Results:**

Lymph node metastasis rate showed no statistically significant difference between the standard 2FLND group and the total 2FLND group (71.1% and 63.3%, respectively, *P* > 0.05). The incidence of postoperative complications was 5.8% (7/121) in the standard 2FLND group, which was lower than that in the total 2FLND group [17.0% (25/147)], with a statistically significant difference (*χ*^2^ = 7.948, *P* < 0.01). The 5-year survival rate of the standard 2FLND group and the total 2FLND group was 29.8% and 28.6%, respectively, without statistically significant difference (*χ*^2^ = 0.005, *P* > 0.05). The lymph node recurrence rate in the standard 2FLND group was 41.3% (50/121), which was higher than 19.0% (28/147) of the total 2FLND group (*χ*^2^ = 15.959, *P* < 0.01).

**Conclusion:**

Compared with standard 2FLND, total 2FLND does not improve the postoperative survival of patients with esophageal carcinoma, and the risk of complications is higher.

## INTRODUCTION

Radical esophagectomy is the preferred treatment method for esophageal carcinoma (EC) 1, and lymphatic metastasis is the main cause for the failure of radical esophagectomy.^2^ Studies have shown that the lymph node metastasis rate of esophageal cancer is as high as 26.2%-37.8%, and the deeper the tumor infiltration, the easier the lymph node metastasis.^3^ However, there is no uniform standard for lymph node dissection (LND) in esophagectomy up to now. Traditional two-field lymph node dissection (2FLND) is commonly used in China. Some researchers^4^ advocate the use of extended 2FLND, but its efficacy is still uncertain. In this study, the clinical data of patients undergoing radical esophagectomy in our hospital from January 2008 to November 2015 were reviewed, and the effects of different LND methods on the clinical efficacy and complications of patients with EC were explored, so as to guide the clinical selection of the optimal LND method.

## METHODS

The clinical data of 268 patients undergoing radical esophagectomy and regular follow-up in our hospital from January 2008 to December 2015 were retrospectively analyzed. All the patients received Ivor-Lewis esophagectomy, including 121 patients undergoing standard 2FLND (standard 2FLND group) and 147 patients receiving total 2FLND (total 2FLND group). All the patients were confirmed as esophageal squamous cell carcinoma (ESCC) by preoperative gastroscope-guided pathological examination. Electrocardiography (ECG), esophagography, and neck, chest and upper abdomen CT examinations were performed routinely. Patients with cervical lymph node metastasis and preoperative neoadjuvant therapy were excluded. No statistically significant differences were found in clinical data including gender, age and lesion site between the standard 2FLND group and the total 2FLND group (*P* > 0.05), as shown in Table-I.

**Table-I:** Comparison in clinical data between standard 2FLND group and total 2FLND group.

Item	Standard 2FLND group (n = 121)	Total 2FLND group (n = 147)	χ^2^/t	P
Gender			1.327	> 0.05
Male	116	136		
Female	5	11		
Age (year)	58.1 ± 10.2	58.6 ± 9.7	0.410	> 0.05
Complications			0.059	> 0.05
Yes	94	116		
No	27	31		
Lesion site			0.259	> 0.05
Upper segment	11	16		
Middle segment	89	107		
Lower segment	21	24		
Gross type			0.675	> 0.05
Superficial type	5	9		
Medullary type	83	102		
Ulcerative type	20	23		
Fungoid type	11	10		
Constrictive type	2	3		
TNM stage			0.405	> 0.05
Ia	5	6		
Ib	7	9		
IIa	16	19		
IIb	55	63		
IIIa	23	31		
IIIb	15	19		
Tumor diameter (cm)	5.3 ± 1.8	5.1 ± 1.9	0.878	> 0.05
Vascular tumor thrombus			0.030	> 0.05
Yes	18	23		
No	103	124		

### Ethical Approval:

The study was approved by the Institutional Ethics Committee of Affiliated Hospital of Hebei University on March 1, 2016, and written informed consent was obtained from all participants

### Methods:

All the patients received Ivor-Lewis esophagectomy and LND. The lymph nodes were grouped according to the AJCC lymph node distribution pattern. The scope of dissection in the standard 2FLND group included: lymph node group 16 under the diaphragm in the upper abdomen, lymph node groups 17, 18, 19 and 20 at the upper edge of pancreas, and lymph node groups 7, 8, 9 and 15 in the chest. On the basis of the standard 2FLND group, lymph node groups 2 and 4 were additionally dissected in the total 2FLND group. All patients were given individualized chemotherapy after surgery.All the patients were followed up by telephone of our department till the end of October 2020, with follow-up duration of 4-86 months (median, 35 months). LND status, postoperative complications, survival rate and lymph node recurrence were statistically compared between the two groups.

### Statistical Analyses:

SPSS19.0 was used for statistical analysis. Survival analysis was carried out using the Kaplan-Meier method and Log-Rank test. The measurement data were expressed as mean ± standard deviation (x̄± s), and compared by the *t* test. The enumeration data were compared using the *χ*^2^ test. *P* < 0.05 was considered as statistically significant.

## RESULTS

A total of 3,675 lymph nodes were dissected in 121 patients of the standard 2FLND group. Among them, 86 patients showed lymph node metastasis by postoperative pathology, with 366 metastatic lymph nodes (lymph node metastasis rate, 71.1%). In the total 2FLND group, a total of 4,516 lymph nodes were dissected from the 147 patients, 93 of which presented lymph node metastasis by postoperative pathology (501 metastatic lymph nodes; lymph node metastasis rate, 63.3%). The lymph node metastasis rate showed no statistically significant difference between the two groups (*P* > 0.05). No statistically significant differences were found in lymph node metastasis rate in the upper abdomen, mid-inferior mediastinum or superior mediastinum between the two groups (*P* > 0.05), as seen in Table-II.

**Table-II:** Comparison in LND status between two groups (%).

Item	Standard 2FLND group (n = 121)	Total 2FLND group (n = 147)	χ^2^/t	P
Lymph node metastasis rate	71.1 (86/121)	63.3 (93/147)	1.825	> 0.05
Lymph node metastasis rate in the upper abdomen	57.9 (70/121)	54.4 (80/147)	0.317	> 0.05
Lymph node metastasis rate in the mid-inferior mediastinum	39.7 (48/121)	34.0 (50/147)	0.915	> 0.05
Lymph node metastasis rate in the superior mediastinum	13.2 (16/121)	20.4 (30/147)	2.409	> 0.05

The incidence of postoperative complications in the total 2FLND group was 17.0% (25/147), which was significantly higher than 5.8% (7/121) in the standard 2FLND group (*χ*^2^ = 7.948, *P* < 0.01). The proportions of patients with postoperative recurrent laryngeal nerve injury and respiratory failure in the total 2FLND group were significantly higher than those in the standard 2FLND group (*P* < 0.01, *P* < 0.05, Table-III).

**Table-III:** Comparison in postoperative complications between two groups [n (%)].

Item	Standard 2FLND group (n = 121)	Total 2FLND group (n = 147)	χ^2^/t	P
Recurrent laryngeal nerve injury	2 (1.7)	25 (17.0)	17.270	< 0.01
Anastomotic leakage	3 (2.5)	2 (1.4)	0.454	> 0.05
Chylothorax	1 (0.8)	2 (1.4)	0.170	> 0.05
Respiratory failure	1 (0.8)	9 (6.1)	5.182	< 0.05

The 5-year survival rate of the standard 2FLND group was 29.8%, with the median survival time of 41 months (95%CI = 37.5-45.8). In the total 2FLND group, the 5-year survival rate was 28.6%, with the median survival time of 42 months (95%CI = 37.1-46.7). The survival showed no statistically significant difference between the two groups (*χ*^2^ = 0.005, *P* > 0.05). The lymph node recurrence rate in the standard 2FLND group was 41.3% (50/121), which was much higher than 19.0% (28/147) of the total 2FLND group (*χ*^2^ = 15.959, *P* < 0.01).

## DISCUSSION

The surgical treatment for EC aims to prolong the survival time and improve the quality of life of the patients.^5^ LND is directly related to the postoperative survival rate of the patients.^6^ Especially in the patients with lower thoracic EC, lymph node metastasis rate is very high, reaching about 40%.^7^ Therefore, the lymph nodes in the lesion area should be thoroughly dissected as far as possible during esophagectomy, in addition to resecting the cancerous esophagus with sufficient length.^8^-^10^ Standard 2FLND, including mid-inferior mediastinal and abdominal lymph nodes, is commonly used by many domestic scholars. However, superior mediastinal and para-recurrent laryngeal nerve lymph nodes, which are prone to metastasis in EC, can not be completely dissected, resulting in that the postoperative 5-year survival rate of EC patients in China is only 30%-35%.^11^ With the understanding of the rule of lymph node metastasis in EC, total 2FLND has been advocated. On the basis of standard 2FLND, superior mediastinal lymph nodes in bilateral recurrent laryngeal nerve are also included in total 2FLND. Some scholars believe that^12^,^13^ total LND expands the scope of dissection, increases the number of dissected lymph nodes, improves the accuracy of postoperative pathological staging and reduces the probability of local recurrence. In our study, the lymph node metastasis rate of the standard 2FLND group was 71.1%, which was higher than 63.3% of the total 2FLND group, but the difference was not statistically significant (*P* > 0.05). There were also no statistically significant differences in lymph node metastasis rate of the upper abdomen, mid-inferior mediastinum or superior mediastinum between the two groups (*P* > 0.05). These results suggest no significant difference in lymph node metastasis rate in EC between standard and total 2FLND. This may be related to the characteristics of lymph node metastasis in EC. Because the lymphatic network between the esophagus and the mediastinum connects each other, lymph node metastasis can be not only continuous, but also bidirectional and skipped.^14^,^15^ However, Liu Liang et al.^16^ have confirmed that total 2FLND can reduce the rate of lymph node metastasis. We believe that although total 2FLND increased the scope and number of dissected lymph nodes, the results of this study were affected in that lymph node metastasis of EC always occurs early, and most patients in this study were locally advanced. However, in terms of long-term efficacy, the postoperative lymph node recurrence rate in the standard 2FLND group was 41.3% (50/121), which was much higher than 19.0% (28/147) of the total 2FLND group. Therefore, the scope of lymph node dissection has a great impact on lymph node recurrence. The scope of standard 2FLND is small, which increases the probability of lymph node recurrence. Wu Changrong et al.^17^ have confirmed that expanding the scope of LND in EC can improve the postoperative 5-year survival rate of the patients. However, our results showed that the 5-year survival rate was 29.8% in the standard 2FLND group and 28.6% in the total 2FLND group. There was no statistically significant difference in the 5-year survival rate between the two groups, indicating that the two LND methods have no obvious effect on the 5-year survival rate of EC patients. Zhang Aimin et al.^18^ proposed that metastatic lymph nodes are an important factor affecting the prognosis of EC. In patients with multi-group and multi-field lymph node metastasis, the tumors have become systemic lesions from local lesions, and surgical resection is still difficult to achieve the desired effect. In this study, although the total 2FLND group presented a lower recurrence rate, there was no difference in the lymph node metastasis rate from the standard 2FLND group, so there was no improvement in the long-term survival rate. It has been reported that^19^ the 5-year survival rate of EC patients with lymph node metastasis after surgical resection is only 12.6%-19.7%, which is much lower than that in our study.

The enlarged scope of LND increased the incidence of surgical complications. Zhang Kun et al.^20^ have confirmed that total 2FLND is likely to cause recurrent laryngeal nerve injury, with an incidence rate of 0.4%-65.0%. In our study, the incidence of postoperative complications in the total 2FLND group was 17.0%, which was significantly higher than that in the standard 2FLND group (5.8%). Moreover, the proportions of patients with recurrent laryngeal nerve injury and respiratory failure in the total 2FLND group were significantly higher than those in the standard 2FLND group. Therefore, the incidence of postoperative complications after total 2FLND is high. This study compares and evaluates the efficacy of standard two-field lymph node dissection and total two-field lymph node dissection in patients undergoing esophageal cancer surgery from the perspectives of postoperative survival rate and complication rate. It provides clinical guidance for the choice of lymph node dissection during esophageal cancer surgery.

### Limitations of this study

It includes small sample size and retrospective nature of the study. The advantages and disadvantages of these two lymph node dissections should also be evaluated more comprehensively from other aspects besides postoperative survival rate and complication rate.

## CONCLUSION

Total 2FLND did not improve the postoperative survival of patients with EC, on the contrary, it brought a higher risk of complications. Therefore, patients with EC should be accurately evaluated before surgery, so as to adopt appropriate dissection methods.

### Authors’ Contributions:

**QG & YL:** Designed this study and prepared this manuscript, and are responsible and accountable for the accuracy or integrity of the work.

**HL &** **HW:** Collected and analyzed clinical data.

**DZ:** Significantly revised this manuscript.
